# Femtosecond Laser Direct‐Write Plasmonic Nanolithography in Dielectrics

**DOI:** 10.1002/smsc.202200038

**Published:** 2022-08-02

**Authors:** Han Zhu, Bo Wu, Mingsheng Gao, Feng Ren, Wei Qin, Saulius Juodkazis, Feng Chen

**Affiliations:** ^1^ School of Physics State Key Laboratory of Crystal Materials Shandong University Jinan 250100 China; ^2^ Department of Physics Center for Ion Beam Application and Center for Electron Microscopy Wuhan University Wuhan 430072 China; ^3^ Optical Sciences Centre Faculty of Science, Engineering and Technology Swinburne University of Technology Hawthorn VIC 3122 Australia

**Keywords:** femtosecond laser direct-write, plasmonic nanolithography, structural color, surface plasmon resonance

## Abstract

Plasmon‐based devices have founded numerous applications in photonics based on optically excited strong near‐field effect at nanoscale. So far, the large‐area fabrication of periodic plasmonic nanostructures is still challenging due to a small write‐field limitation of lithography‐based techniques, especially for metallic substrates. A novel strategy is proposed to fabricate millimeter‐sized patterns of periodic plasmonic nanostructures inside dielectric materials (glass) through a femtosecond laser direct‐write plasmonic nanolithography approach. Noble metal nanoparticles are formed by the ion implantation in glass and reshaped into a nanowire‐like nanoparticles’ assembly by direct writing with femtosecond laser harnessing plasmonic interaction. As‐designed patterns at nanoscale are inscribed by this type of plasmonic lithography to form wires composed of nanoparticles. Examples for applications, the linear dichroism response, and structural color have been achieved by the plasmonic nanogratings buried inside glass (i.e., at subsurface regions). The work opens a new avenue to manipulate metallic nanoparticles in solids by direct plasmonic nanolithography and offers a reliable implementation of large‐area fabrication of plasmonic nanostructures for diverse range of photonic applications.

## Graphical abstract

This work opens a new avenue to manipulate metallic nanoparticles in solids at the nanoscale via femtosecond laser‐excited interacting plasmons. Polarization‐dependent plasmonic modes and structural coloring are achieved by nanowire‐like particles’ assembly. This approach can be extended to other dielectric functional materials, enabling large‐area direct nanofabrication of low‐cost, compact plasmonic photonic devices toward versatile applications.

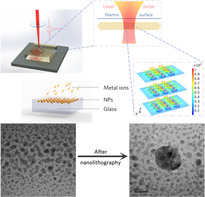

## 1 Introduction

Noble metallic (e.g., silver or gold) nanostructures exhibit extraordinary optical properties due to their surface plasmon resonance excited by electromagnetic waves.^[^ ^1^, ^2^, ^3^, ^4^, ^5^ ^]^ Localized surface plasmon resonance (LSPR) of metallic nanoparticles (NPs)—quantized conduction band electron collective oscillations confined to the nanoscale—with highly localized near‐fields is widely used in surface‐enhanced Raman scattering (SERS),^[^ ^6^, ^7^, ^8^ ^]^ biochemical sensing and detection by surface‐enhanced IR absorption (SEIRAS),^[^ ^9^ ^]^ miniaturized optical devices,^[^ ^10^, ^11^ ^]^ and data storage.^[^ ^12^, ^13^ ^]^ In intricate nanostructures, e.g., periodic arrays of NPs, the coupling between adjacent NPs provides wavelength‐shifted (red/blueshifted) plasmon resonance modes for subwavelength devices in comparison to isolated individual NPs due to coupling.^[^ ^14^ ^]^ Recent advances in plasmonic hybridization theory also elucidate the analogy between plasmons in complex nanostructures and electrons that form molecular orbitals,^[^ ^15^, ^16^, ^17^ ^]^ which offers insights into the use of NPs as “plasmonic atoms” to assemble nanostructures with customizable properties. So far, several top‐down or bottom‐up processing routes, such as focused ion beam (FIB) milling,^[^ ^18^ ^]^ electron beam lithography (EBL),^[^ ^19^ ^]^ and self‐assembly,^[^ ^20^, ^21^ ^]^ have been utilized to produce tailored plasmonic nanostructures composed of NPs. However, inherently small write fields of FIB and EBL lead to obvious obstacles of large‐area (with cross sections larger than 1 mm) nanofabrication due to the long processing time and difficulty of high‐precision stitching of individual write fields. Self‐assembly requires large‐size high‐resolution nanomasks with a prohibitively high cost. In addition, these techniques are limited to fabricate nanostructures on the surface, and are unable to define NPs inside solids where they can be more chemically/environmentally stable. Femtosecond laser direct writing has become a well‐developed technique to produce micro/nanostructures inside glass and other dielectrics for diverse photonics applications.^[^ ^22^, ^23^, ^24^, ^25^ ^]^ It has been successfully applied to form nanoscale gratings and pores through the rapid redistribution of atoms at the focal volume where the laser directly interacts with the target matrix.^[^ ^26^, ^27^ ^]^ Moreover, several studies have reported precipitation or reshaping of NPs at nano/microscale by femtosecond laser‐induced ion reduction or migration.^[^ ^28^, ^29^, ^30^ ^]^ These results demonstrate great potential for direct‐write nanolithography of plasmonic NPs through high‐resolution femtosecond laser processing. Nevertheless, controllable and large‐area fabrication of nanopatterns in solids is still challenging, and the direct nanolithography of plasmonic NPs in glass for tailored nanostructures has not been realized.

In this article, we propose a novel strategy to fabricate plasmonic nanostructures by using femtosecond laser direct‐write plasmonic nanolithography. The Au/Ag NPs are incorporated into glass as encapsulated plasmonic dots, which generated LSPR upon irradiation by focused femtosecond laser beams causing local thermal growth/sintering/diffusion of NPs and their clusters. The near‐field localization from LSPR enables induced migration and reshaping of NPs in glasses at submicrometric scale. The femtosecond laser direct‐write nanolithography for plasmonic NPs processing is then realized by adjusting the laser power, repetition rate, and scanning velocity. Large millimeter‐scale areas are fabricated with polarization‐dependent plasmonic modes (a linear dichroism) and small‐size effects of nanostructures (structural color) are achieved by assembly of plasmonic NPs periodic arrays.

We use the ion implantation technique to incorporate Au/Ag elements into commercially available fused silica (SiO_2_) or BK7 glass samples (Table S1, Supporting Information). Ion implantation offers a widely applicable route to encapsulate metallic NPs in any glass substrates regardless of their matrix and composition.^[^ ^31^, ^32^, ^33^ ^]^ The implanted Ag or Au ions exist in the glass as ion/atom impurity or NPs without further reduction process, while the size and dielectric environment of NPs can be well controlled by selecting appropriate parameters for implantation. The Ag or Au NPs form a thin layer containing plasmonic clusters inside glass located at the depth of ≈100 nm from surface, which is determined by the ionization state of ions and acceleration voltage. Figure S1–S5, Supporting Information, depict the detailed information of the NPs in glass by ion implantation.

## 2 Results and Discussion

### 2.1 Plasmonic Nanolithography

**Figure 1:**
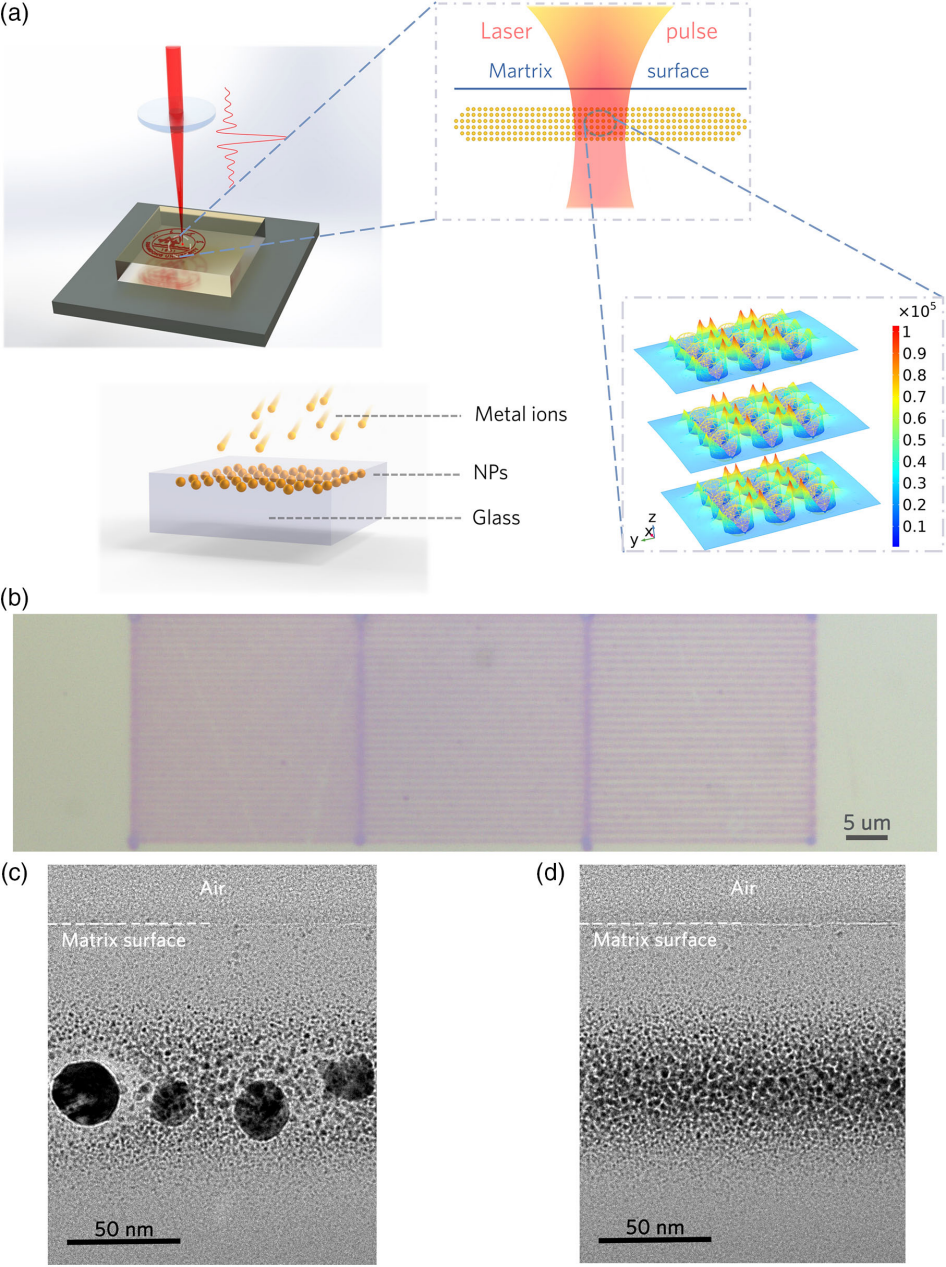
Femtosecond laser direct nanolithography of Au NPs inscribed inside fused silica. a) Schematic of the ion implantation process and femtosecond laser writing of Au NPs in fused silica system. Au ions are implanted into the glass and aggregate to Au NPs as the ion concentration exceeds the threshold of solubility. Inset: near‐field distribution of Au NPs under 1030 nm light excitation. b) Top optical microscope view of laser inscribed Au nanowire arrays buried in fused silica. The writing speed is 4 mm s^−1^, and the period is 700, 800, and 900 nm from left to right. c,d) Cross‐sectional TEM images of the laser‐irradiated (c) and unirradiated (d) of the Au NPs in fused silica. The cross section is perpendicular to the laser writing direction. Clear Au NPs modification by the femtosecond laser is observed, while the glass surface remains intact after the laser writing.

In **Figure** **1**a, we illustrate the process of femtosecond laser direct‐write plasmonic nanolithography of NPs. The LSPR excited by femtosecond laser generates a strong near‐field around the particle surface. The ultrafast electron emission and nonlinear absorption process induced by the strong local field leads to the temperature accumulation in the glass causing phase transition (melting) in the closest proximity to the NP. For isolated NPs this heat‐affected zone is only limited to several nanometers that is incapable to cause migration of a NP.^[^ ^34^ ^]^ Our previous studies showed that reducing the spacing between NPs significantly enhances the near fields due to the coupling of adjacent plasmon in NPs.^[^ ^35^, ^36^ ^]^ In this work, the size and spacing (down to 1 nm) of NPs are controlled by selecting an appropriate ion fluence/dose.

Figure 1b shows nanowire arrays (i.e., nanogratings with periods of 700, 800, and 900 nm) in fused silica with segregated Au NPs directly inscribed by a femtosecond laser with the central wavelength of 1030 nm and pulse duration of 350 fs. The fabrication process can be seen in Video S1, Supporting Information. The rate exceeding 4 mm s^−1^ is suitable for large‐area photonic device fabrication, which can be further improved by optimizing parameters of laser writing. The power of the femtosecond laser is well below the threshold for apparent glass modification without embedded NPs, indicating that the nanowire formation relies on excited LSPR by ultrafast laser pulses (Figure S6, Supporting Information). The LSPR can be stably excited in a variety of dielectric environments. Figure S7, Supporting Information, presents the realization of similar traces in BK7 glass. From transmission electron microscopy (TEM) images (Figure 1c,d), one can see that the Au NPs are reshaped inside the glass after laser irradiation, and the mean particle size of the NPs increased from 2.91 to 3.06 nm. The minimum width of nanowires can be controlled down to ≈300 nm, which is significant improvement of modification precision compared with usual ultrafast laser‐induced NPs.^[^ ^28^, ^29^, ^30^, ^34^ ^]^ High‐resolution transmission electron microscopy (HRTEM) and energy‐dispersive X‐ray spectroscopy mapping further confirm the migration and particle size changes of Au NPs during lithography (Figure S8, S9, Supporting Information), which originates from the local softening of the matrix around the particles induced by the strong near‐field of high‐density Au NPs. This is known as the Ostwald ripening. The numerical simulations of uniformly distributed multilayer NP arrays (Figure S10, Supporting Information) reveal near‐field of Au NPs under 1030 nm. Notably, from Figure 1c,d, the boundary of air and sample surface remains smooth after femtosecond laser writing, exhibiting unique advantage of plasmonic lithography acting only on thin NPs layers without damaging the glass surface. This feature is crucial for manipulation of NPs in solids without damaging of dielectric substrates. We also observe no asymmetry related to the writing direction or polarization of the beam in the nanowire inscription, which is consistent with femtosecond laser lithography of nanocrystals in dielectrics.^[^ ^28^ ^]^

**Figure 2:**
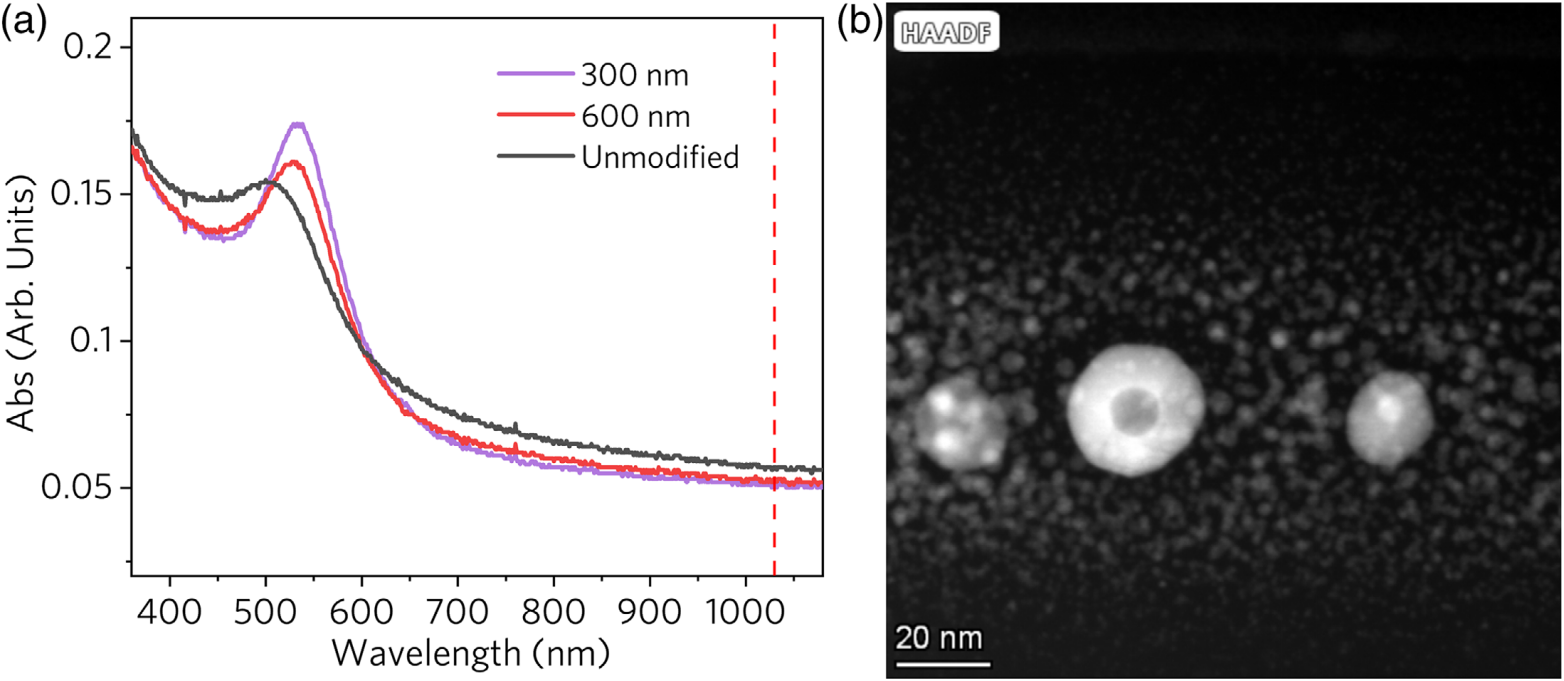
Optical extinction spectra of Au NPs and HAADF image of Au nanoshell structure in fused silica. a) Measured extinction spectra of unmodified NPs and laser‐induced NP arrays with a period of 300 and 600 nm, respectively. The red dashed line indicates the central wavelength (1030 nm) of the femtosecond laser. b) The HAADF image of the cross section of the Au NPs after laser irradiation, corresponding to a writing speed of 10 μm s^−1^.

The femtosecond laser direct‐write nanolithography depends on the LSPR excitation and absorption at Au NPs. On the other hand, the femtosecond pulses modify the Au NPs as well. The morphology change of NPs is reflected by a modified optical absorption spectrum (**Figure** **2**a). After the laser processing, the diameters of Au NPs increase, which corresponds to a redshift of the extinction peak in respect to that of unmodified NPs. Plasmons are excited at 1030 nm (red line in Figure 2a), far from the LSPR absorption peak (≈500 nm), which allows the heat accumulation at the particles’ location to soften the glass matrix while unable to melt the Au NPs. In addition, the stronger LSPR effect from the NPs after migration and reshaping could lead to further size and shape evolution of NPs. Figure 2b shows the high‐angle annular dark field (HAADF) image of the Au nanoshell structures, which is due to the photothermal reconstruction of a diffusion of free‐electron‐driven transport during plasmon resonance.^[^ ^37^ ^]^

### 2.2 Nanowire‐like NPs’ Assembly for Photonics Applications

**Figure 3:**
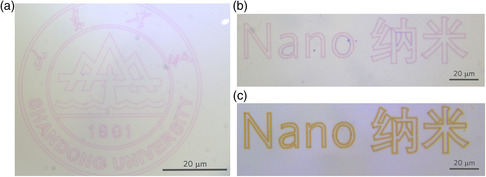
Demonstration of tailored patterning and laser nanolithography of Au or Ag NPs in fused silica. a) Logo of Shandong University composed of Au NPs in glass. b,c) Logos of “Nano” and its Chinese characters composed of Au or Ag NPs.

The nanowires are elementary structures which could be used to form/build more complex tailored patterns. We demonstrate this capability by directly writing logos of Shandong University and “Nano” composed of Au NPs inside glass (**Figure** **3**a,b). All these patterns are inscribed in fused silica by the plasmonic nanolithography of Au NPs from the computer‐generated graphic files. To assess whether the observed nanolithography plasmonic NPs are limited to the case of gold, or whether this phenomenon can also be manifested in other noble metal elements, we perform a similar study in Ag NPs embedded fused silica (see Experimental Section and Table S1, Supporting Information). The pattern of Ag NPs is yellow due to the resonant absorption of Ag NPs (at ≈438 nm) different from that of Au NPs (Figure 3c). Supplementary Section 2 presents the nanograting composed of Ag NPs and the corresponding TEM image. Figure 3 and S7d, Supporting Information, demonstrate that arbitrary geometric patterns can be directly inscribed as assembly of corresponding plasmonic NPs and laser‐induced tracks can cross each other without any distortion.

**Figure 4:**
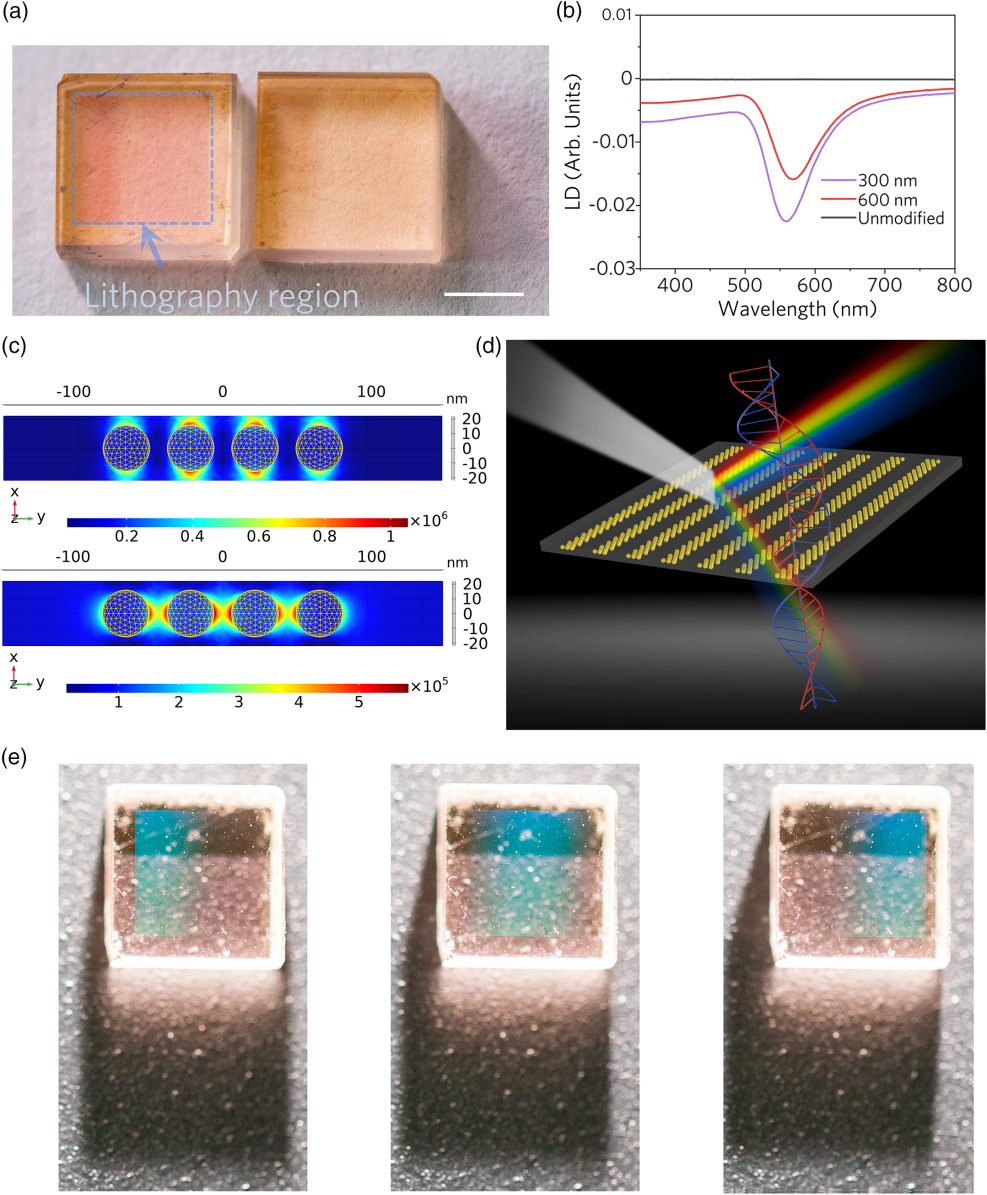
Line dichroism and structural color of subwavelength nanograting of Au NPs in fused silica. a) Image of a 600 nm‐period grating composed of Au NPs in a 3.5 × 3.5 mm^2^ region and unmodified sample under visible light illumination. Scale bar: 2 mm. b) Measured linear dichroism of the unmodified sample and nanogratings. c) Simulated localized surface plasmons of periodic nanogratings with different near‐field coupling modes. LSPR is excited by light (600 nm) propagating along the *z*‐axis, corresponding to longitudinal modes polarized along the *x*‐axis and transverse modes polarized along the *y*‐axis. d) Schematic illustration of the simultaneous realization of linear dichroism and structural color in one plasmonic system. e) Camera‐captured structural color of nanogratings with a period of 600 nm. With the movement of the light source, the stable structural color in the 3.5 × 3.5 mm^2^ region is clearly observed.

The subwavelength assembly of noble metallic NPs enables realization of customized plasmonic patterns using femtosecond laser nanolithography for practical photonics applications. For example, in particle chains composed of closely arranged NPs, the coupling effect of interparticle electromagnetic fields not only enhances the near field, but leads to a resonance frequency shift that depends on the polarization direction of the excitation light, which can be employed to manipulate photon polarization at the nanoscale.^[^ ^38^, ^39^ ^]^ This opens ultrafast light‐by‐light control of transmission of polarized light in the presence of a cross‐polarized field. Through wire‐by‐wire writing, we construct nanograting structures composed of Au NPs with different periods (300 and 600 nm) in area of 3.5 × 3.5 mm^2^ in fused silica (**Figure** **4**a). The linear dichroism response is observed in both nanogratings, and as the period reduces, the dichroism effect strengthens (Figure 4b). It should be pointed out that the linear dichroism response at 300 nm period also confirms the possibility of achieving nanowires with width below 300 nm by the plasmonic nanolithography approach. Figure 4c indicates the polarization‐dependent plasmons due to the different excitation modes, which is revealed by the numerical simulations based on the finite element method (see Experimental Section for the modeling of NP chains in glass). The absorption and extinction spectra of different plasmonic resonance modes are shown in Figure S12, Supporting Information. The longitudinal excitation mode causes a redshift (intra‐NP coupling) of the resonance for vertical polarization and, in turn, the transverse excitation mode brings out a blueshift for parallel polarization (reduced coupling).

As shown earlier,^[^ ^40^ ^]^ the periodic NP arrays obtained by the proposed inscription are also capable of producing vibrant and tunable structural colors. Figure 4e, S13, Supporting Information, and Video S2, Supporting Information, show the angle‐dependent structural color captured by the photo/video camera. Compared with surface textures, the structural colors originating from the Au NPs encapsulated glass possess high stability and have conceivable potential for photonic integration. In addition, as the interference and diffraction from a grating are strongly affected by the period when it is close to the nanowire width, no structural color is observed for a period of 300 nm. This also implies that, with precise control of the inscribed patterns and spacing of individual nanowires, the linear dichroism and structural color could be tailored independently of each other in a same plasmonic system (Figure 4d).

## 3 Conclusions

In summary, we propose a femtosecond laser direct‐write plasmonic nanolithography to realize assembly of noble metallic NPs inside glasses. The plasmon‐induced optical absorbance and localization of the near fields around NPs enable direct inscription of plasmonic nanowires with width below the diffraction limit of 1030 nm wavelength femtosecond laser beam. The migration and reshaping of metallic NPs in an all‐solid environment has been newly presented for implanted ions, which play a crucial role in fabrication of more intricate patterns. The polarization‐dependent plasmonic modes and structural coloring have been achieved by nanogratings composed of Au NPs, which is promising for novel multidimensional optical storage and information encoding techniques. In addition, this approach can be extended to other dielectric functional materials, enabling large‐area direct nanofabrication of low‐cost, compact plasmonic photonic devices toward versatile applications. As implantation is depth selective, nanoscale layered patterns can be formed with laser heating restores glass structure/network.

## 4 Experimental Section

4.1

4.1.1

##### Sample Fabrication

Commercial fused silica (SK1300, Ohara Inc., Japan) and BK7 glass (N‐BK7, SCHOTT AG., Germany) wafers with a size of 10 × 10 × 2 mm^3^ were optically polished for ion implantation. The Au^+^ and Ag^+^ ions with energies of 160 and 80 keV, respectively, were implanted into the glass substrate by ion‐implanter LC22‐1C0‐01 at Wuhan University. The ion fluence of the corresponding ions for each sample is 3 × 10^16^ ions cm^−2^. The noble metal ions are randomly embedded in the glass, and through ionization induced in the solid during implantation, the charge state of the implants can be accommodated to the local environment in the matrix. When the ion/atom concentration exceeds the solubility limit, they aggregated to form NPs by thermal activation of their growth. After implantation, the samples were segmented by a saw‐dicing for further laser direct writing.

We used a fiber chirped pulse amplification (FemtoYLTM‐25, YSL Photonics Ltd., China) system with a center wavelength of 1030 nm and a duration of 350 fs. The laser repetition rate was set to 500 kHz and 5 MHz in the experiments, and qualitatively consistent results were also found at 1 and 2 MHz. The laser was focused below the sample surface by a 0.45 numerical aperture (NA) objective lens with magnification of 50×. The diameter of the laser beam at the focus was about 2 μm (1.22*λ*/NA). A half‐wave plate combined with a polarizing beam splitter was used to control the laser power. A computer‐controlled 6‐axis motorized stage (Hybrid Hexapod, Alio Industries Inc., USA) with a spatial resolution of 0.1 μm was used to manipulate the working position and displacement velocity of the sample. Line scan speed was typically 10 μm s^−1^ at 500 kHz repetition rate and 4 mm s^−1^ at 5 MHz repetition rate. All femtosecond laser processes were performed at standard atmospheric pressure and room temperature.

##### Sample Characterization

Optical microscopic images of laser nanolithography regions were captured by a self‐made imaging system equipped with a CCD camera mounted on a femtosecond laser processing platform. TEM measurements are performed by Talos F200X G2 (S)TEM (Thermo Fisher Scientific Inc., USA) and JEM‐2100 (JEOL Ltd., Japan). The linear extinction spectra of the samples in the wavelength range of 350–1100 nm were measured with a UV–vis–NIR spectrophotometer (U‐4100, Hitachi Ltd., Japan) at room temperature. Linear dichroism spectra were performed by using Chirascan V100 (Applied Photophysics Ltd., UK). The scanning step of the linear dichroism was 1 nm (0.5 s per point) with a bandwidth of 1.5 nm. The morphological features and structural color of the sample under visible light illumination were captured by a single‐lens reflex camera (Canon EOS 200D, Canon Inc., Japan). For reflective structural color capture, the aperture value is f/28, the exposure time is 1/100 s, and the ISO‐1600, and for transmission structural color capture, the aperture value is f/5.6, the exposure time is 1/15 s, and the ISO‐3200.

##### Numerical Simulation

Numerical simulations of the optical properties of NPs, such as near‐field distribution and absorption cross section, were performed using the commercial software COMSOL Multiphysics. Based on the actual NP distribution, a triple‐layer NP model and a periodic NP chain model were established to demonstrate the near‐field enhancement and polarization‐dependent plasmon resonance caused by LSPR coupling, respectively. The electromagnetic waves were incident vertically (along the *z*‐axis) from the air to the glass embedded with NPs. Depending on the particle size, the refractive index of Au NPs was defined by the Lorentz–Drude model for the triple‐layer NP model, and the Brendel–Bormann model for the nanochain model. Periodic port boundary conditions and periodic boundary conditions are applied to the boundary perpendicular to the direction of light propagation and other boundaries, respectively. For the triple‐layer NP model, perfectly matched layers were applied around the physical region to simulate a domain with open boundaries.

## Conflict of Interest

The authors declare no conflict of interest.

## Supporting information

Supplementary Material

## Data Availability

The data that support the findings of this study are available from the corresponding author upon reasonable request.
