# Femtosecond Laser Direct‐Write Plasmonic Nanolithography in Dielectrics

**DOI:** 10.1002/smsc.202300054

**Published:** 2023-05-16

**Authors:** Han Zhu, Bo Wu, Mingsheng Gao, Feng Ren, Wei Qin, Saulius Juodkazis, Feng Chen

1

*Small Sci.* **2022**, *2*, 2200038

DOI: 10.1002/smsc.202200038

A funder was missing from the acknowledgements section of the originally published article: National Key Research and Development Program of China (Grant No. 2019YFA0705000). The corrected acknowledgements section reads:

This work was supported by National Key Research and Development Program of China (Grant No. 2019YFA0705000); National Natural Science Foundation of China (NSFC) (grant no. 11535008); Natural Science Foundation of Shandong Province (grant no. ZR2021ZD02); and Project 111 of China (grant no. B13029).

