# Supplementary material for: Femtosecond Laser Direct‐Write Plasmonic Nanolithography in Dielectrics
Source: Small Sci. 2022 Aug 2;2(9):2200038. doi: 10.1002/smsc.202200038 (PMC11935867; doi:10.1002/smsc.202200038)
Supplement: Supplementary file 1 — Supplementary Material [file SMSC-2-2200038-s001.zip › Supporting information-R1.pdf]

Supporting Information

Femtosecond-laser direct-write plasmonic nanolithography in dielectrics

*Han Zhu, Bo Wu, Mingsheng Gao, Feng Ren, Wei Qin, Saulius Juodkazis, and Feng Chen**

This file includes:

Supplementary Text

Figs. S1 to S13

Tables S1

Other Supplementary Materials for this manuscript include the following:

Videos S1 to S2

Supplementary Section 1: Preparation and characterization of NPs encapsulated in glass

The Au/Ag NPs inside glass (sub-surface) were fabricated by ion implantation, with the detailed preparation parameters are shown in **Table S1** followed by femtosecond laser inscription. The range and distribution of ions in fused silica glass were simulated by the Stopping and Range of Ions in Matter (SRIM) code as shown in **Figure S1**[1]. **Figure S2** and **S3** show the distribution and morphology of NPs (unmodified) in the glass after implantation. Figure S2 shows high-resolution transmission electron microscopy (HRTEM) images and electron energy scattering X-ray spectra of Au NPs (corresponding to Figure 1d) formed inside fused silica. It can be seen that small size and closely spaced NPs were fabricated for the next step of femtosecond laser direct writing.

Due to the different ion behaviors and implantation parameters, larger particles with small spacing were obtained for Ag NPs as shown in Figure S3. **Figure S4** shows the particle diameter distribution of NPs obtained by statistical analysis of TEM images. The measured linear optical extinction spectra of sample 2 is shown in **Figure S5**.

For the case of Au ion implantation into BK7 glass, please refer to our previous work[2].

Table S1. Parameters of sample preparation.

No.	Implanted ions	Fluence [ions cm^{-2}]	Energy [keV]	Glass
1	Au^+	3×10^{16}	160	SiO_2
2	Au^+	3×10^{16}	160	BK7
3	Ag^+	3×10^{16}	80	SiO_2

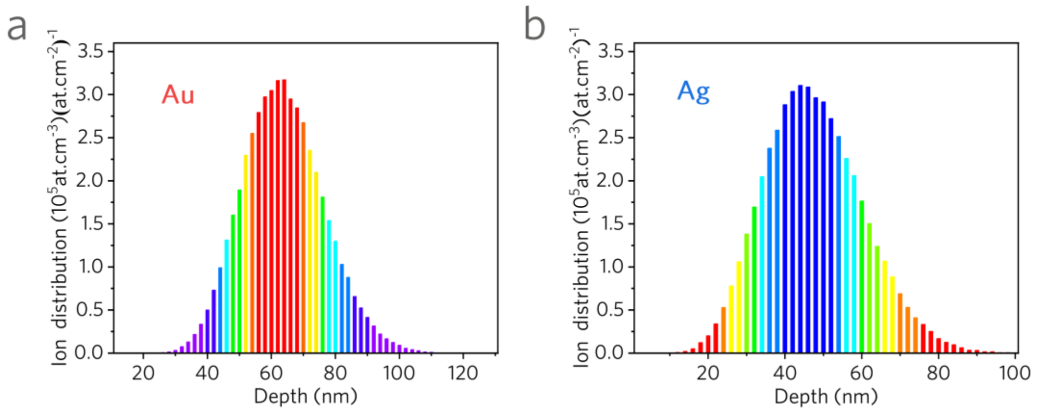

Figure S1. Ion distribution of different elements in fused silica calculated by SRIM: a) Au and b) Ag.

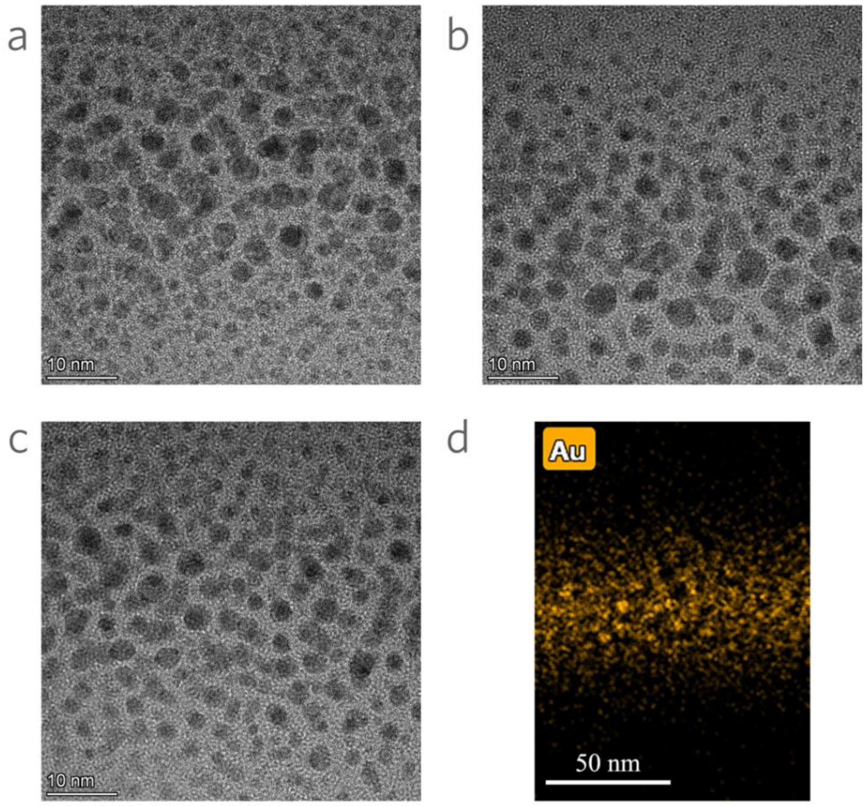

Figure S2. Morphological characterization of Au NPs embedded inside fused silica. a-c) Cross-sectional HRTEM images of sample 1. d) Element mapping of the NPs layer in sample 1 after ion implantation.

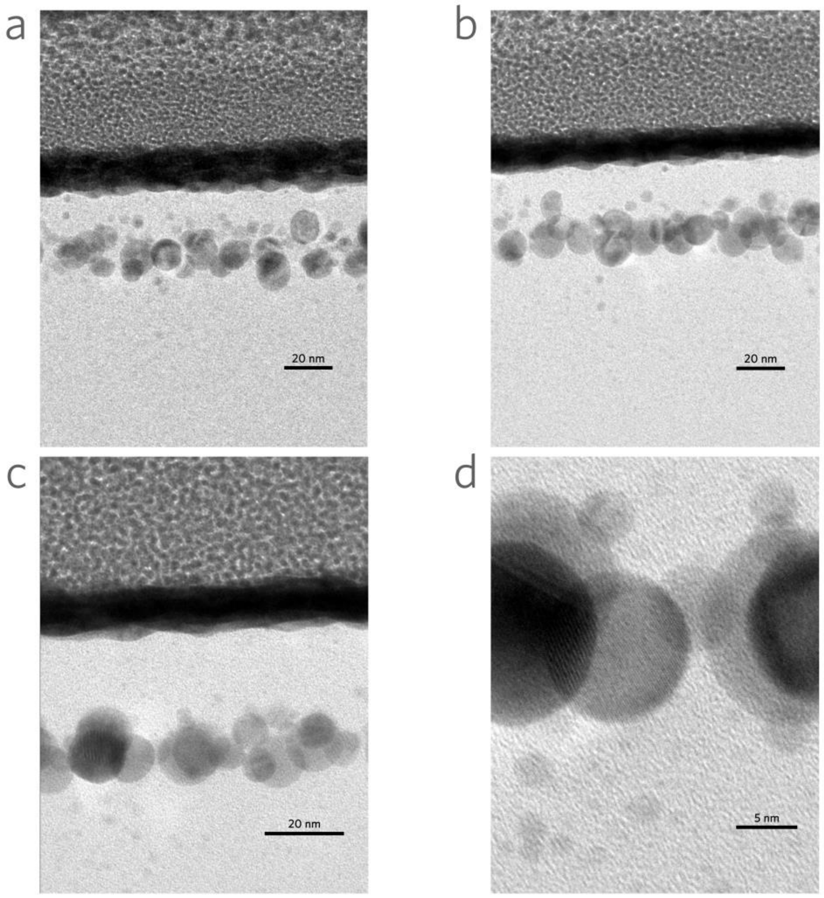

Figure S3. Morphological characterization of Ag NPs embedded inside fused silica. a-c) Cross-sectional TEM images of sample 2. d) HRTEM images of the NPs layer in sample 3.

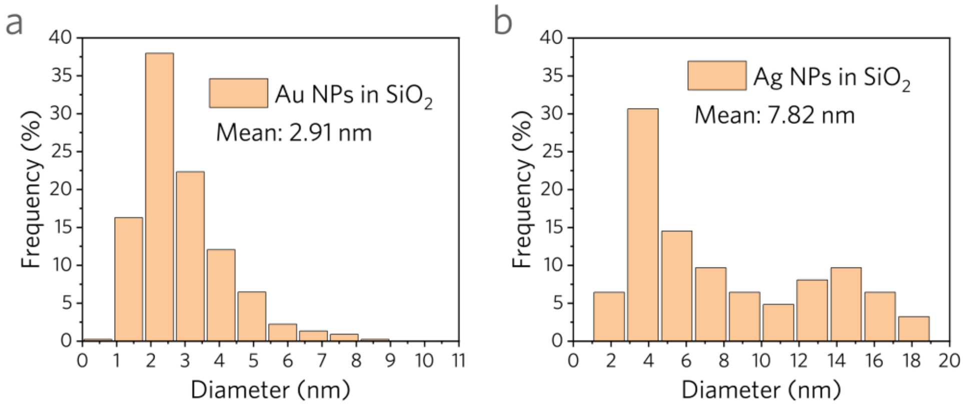

Figure S4. Diameter distribution of Au/Ag NPs in fused silica based on TEM images.

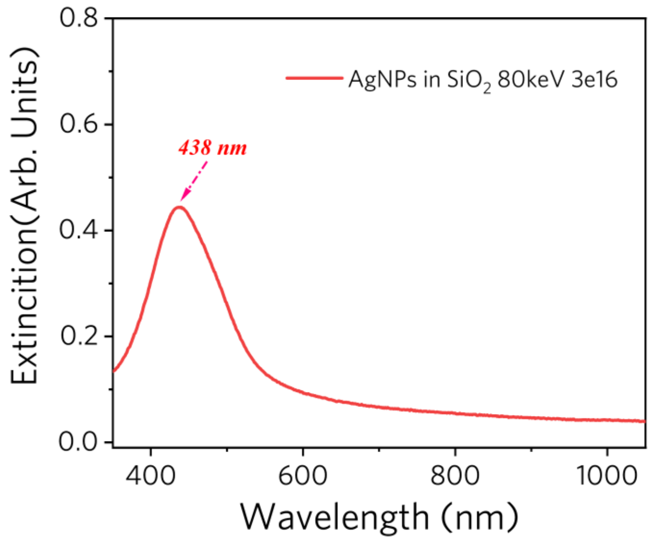

Figure S5. Measured experimental extinction spectrum of sample 3.

Supplementary Section 2: NPs migration and reshaping by femtosecond laser direct writing and plasmon excitation.

In this work, a femtosecond laser with the central wavelength of 1030 nm and pulse duration of 350 fs was used to directly inscribe modified regions inside samples. The laser pulse energy was fixed at ~3.5 nJ for the formation of nanowire arrays. Therefore, the average laser power depends on the repetition rate, with an average laser power of ~170 mW for the repetition rate of 5 MHz and ~17 mW for the repetition rate of 500 kHz.

We performed similar experiments on the backside of sample 1 (fused silica glass without Au NPs) as shown in **Figure S6**. It can be seen that the average laser power used for plasmonic nanolithography was well below the threshold for clear modification of pure glass.

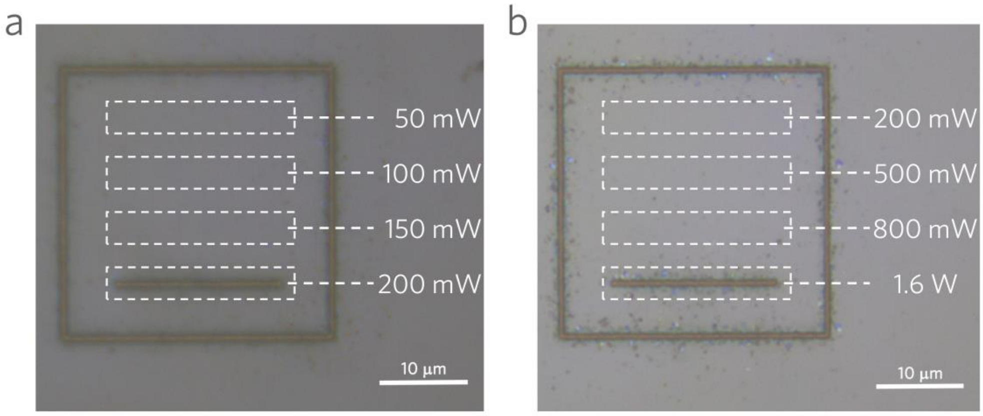

Figure S6. Femtosecond laser direct writing of pure fused silica glass at different repetition rates and average laser power: a) 500 kHz and b) 5 MHz.

Figure S7 summarizes the femtosecond laser direct-write plasmonic nanolithography of different samples. Figures S7a-c shows that nanowire-like nanoparticle assembly which can be realized in both BK7 and SiO_2, while the laser-induced modification traces were crisscrossed with each other without any distortion. The top optical microscope view of laser inscribed Ag nanowires buried in fused silica is shown in Figure S7d.

To further demonstrate the migration and reshaping of NPs in the laser-irradiated region, we performed TEM characterization of the Au nanowire arrays after femtosecond laser direct writing, as shown in **Figure S8** and **S9**. From the comparison of Figure S8,S9 with S4, it could be noted that the mean size of NPs does not increase significantly after laser action, which can be explained by: (1) a portion of NPs migrated and reshaped to form larger particles, many smaller NPs remained in the modified region without changes, and (2) the strong near field excited by the laser beam reduced a solubility limit of the modified region causing a local softening of the matrix around the particle. The later was essential for precipitation of implanted ions/atoms to form smaller NPs[2-4].

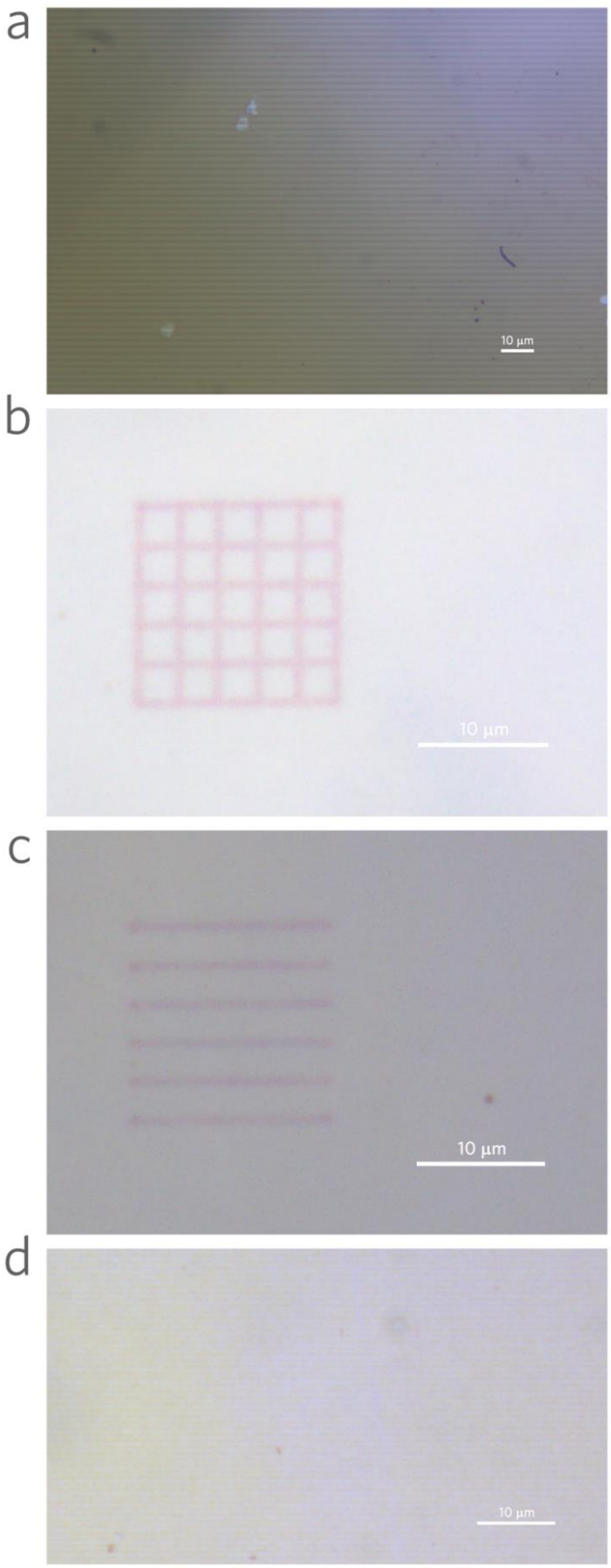

Figure S7. (Top) An optical microscope view of laser inscribed nanowire arrays composed of NPs buried in different glasses: a,b) Au NPs in fused silica. c) Au NPs in BK7 glass. d) Ag NPs in fused silica. The laser repetition rates were (a) 500 kHz and (b-d) 5 MHz, respectively.

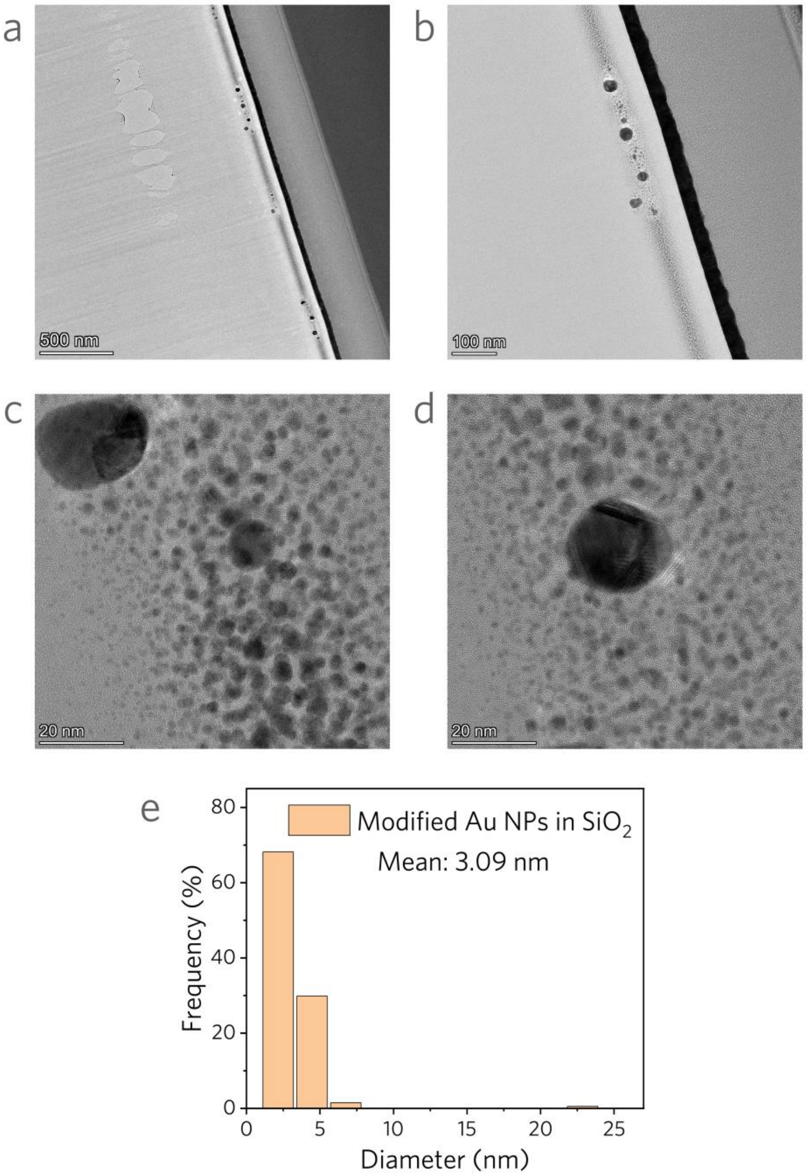

Figure S8. Morphological characterization of AuNPs in Sample 1 after laser irradiation at repetition rate of 5 MHz. a,b) Cross-sectional TEM images. c,d) HRTEM images of the laser irradiated region. e) Diameter distribution of Au NPs after laser irradiation.

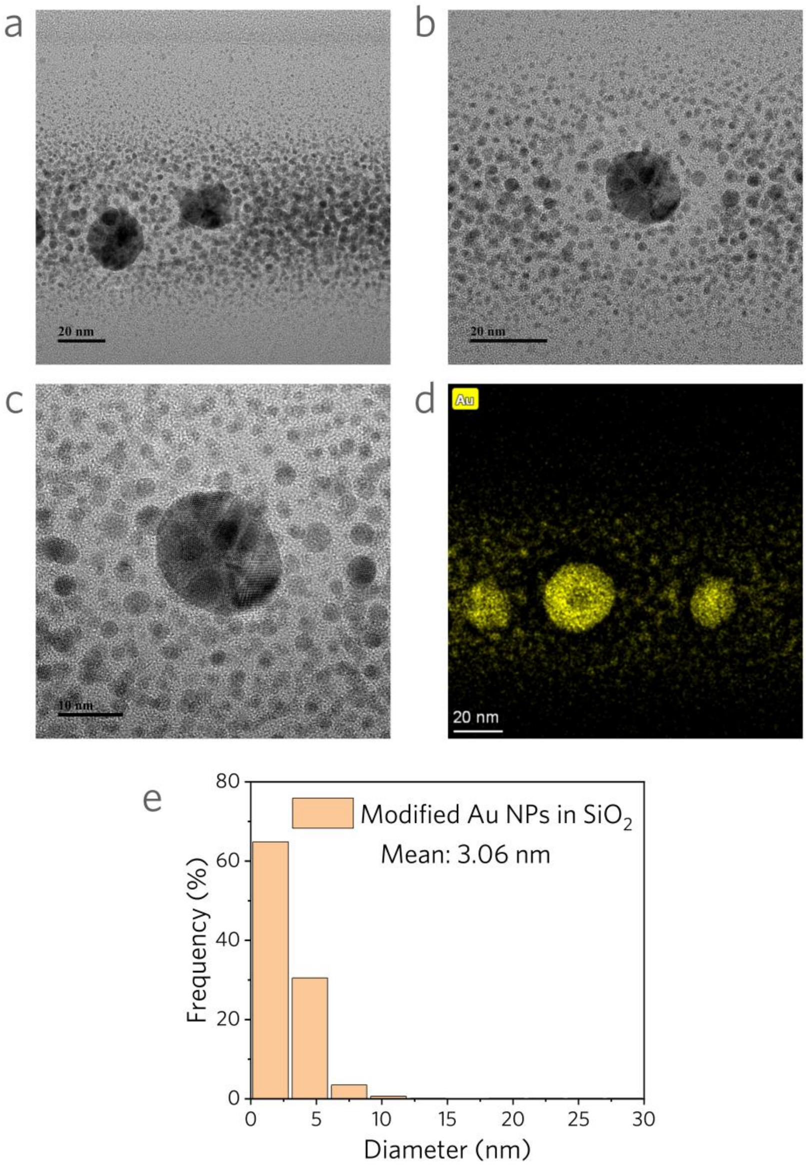

Figure S9. Morphological characterization of AuNPs in Sample 1 after laser irradiation with a repetition rate of 500 kHz. a-c) Cross-sectional TEM and HRTEM images of the laser irradiated region. d) Element mapping of the NPs layer. e) Diameter distribution of Au NPs after laser irradiation.

The near-field distribution of Au NPs under 1030 nm light excitation simulated by the finite element method is shown in **Figure S10**. It is obvious that compared with isolated NPs, the coupling between NPs LSPR significantly enhanced the near-field, while the high density of plasmonic dots also contributed to the local softening of the matrix around the particle. **Figure S11** shows the TEM characterization of the cross-section of laser inscribed Ag nanowire arrays. Femtosecond laser-induced NPs reshaping and migration were also observed in Ag ion-implanted fused silica. Unlike the case of Au, the lower density and larger particle spacing of Ag NPs in sample 2 resulted in less obvious changes in particle morphology after plasmonic nanolithography.

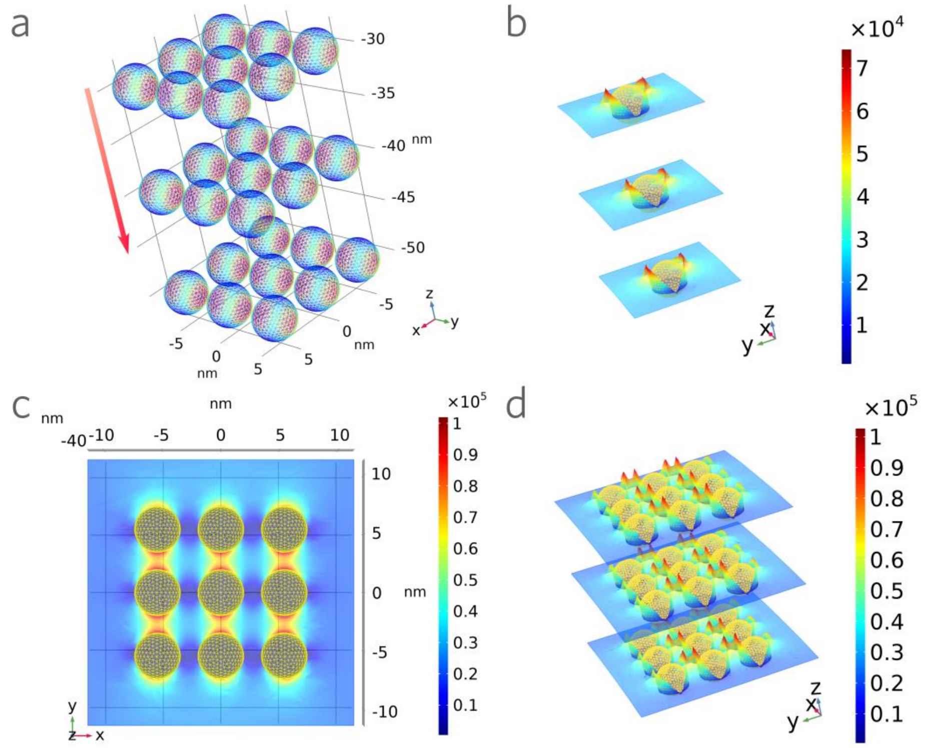

Figure S10. a) Triple-layered nanoparticles' array defined strong near-field, where localized surface plasmons are excited by light (1030 nm). which propagated along the *z*-axis and was linearly polarized along *y*-axis. b) LSPR of isolated triple-layered particles. c,d) Near-field distribution of adjacent plasmon-coupled monolayer and multilayer NPs**.**

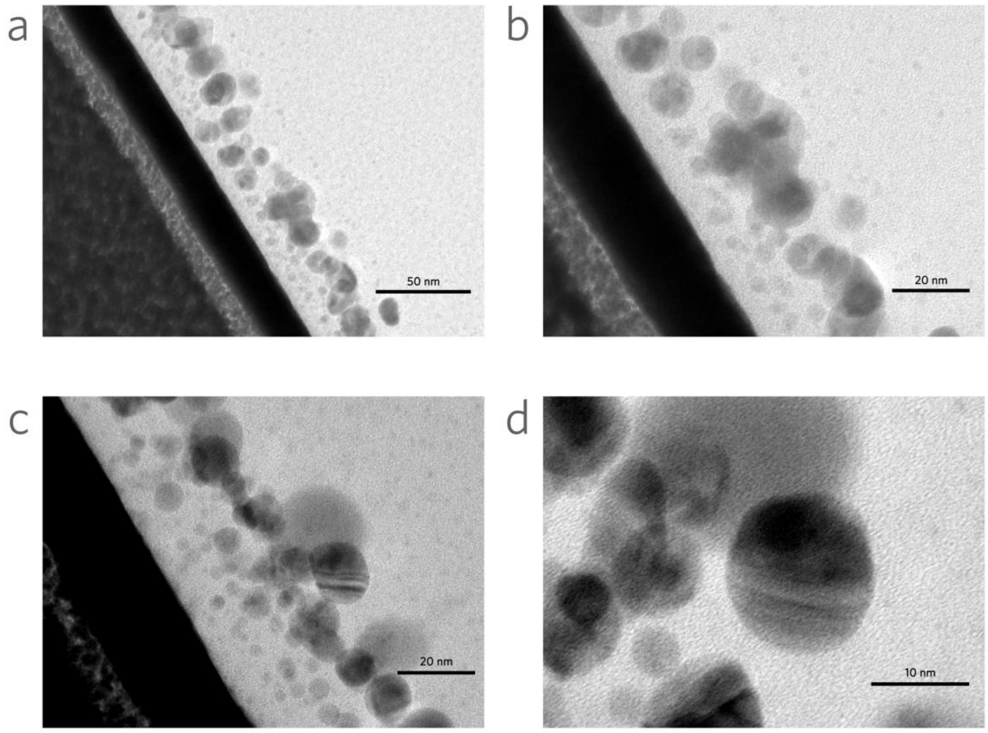

Figure S11. Morphological characterization of Ag NPs in Sample 3 after laser irradiation with a repetition rate of 5 MHz. a-c) Cross-sectional TEM images. d) HRTEM images of the laser irradiated region.

Supplementary Section 3: Polarization-dependent plasmon resonance modes and structural colour.

The absorption and extinction properties of NPs in different plasmonic resonance modes obtained by finite element simulation are shown in **Figure S12**. Depending on the polarization direction of the incident light, the longitudinal excitation mode corresponds to stronger absorption and scattering than the transverse mode.

Not limited to the reflection case, **Figure S13** presents the vibrant transmitted structural colour of the sample as captured by camera. With the movement of the light source (Figure S13a-c), distinct structural colors can be observed throughout the lithography region (Figure 4a).

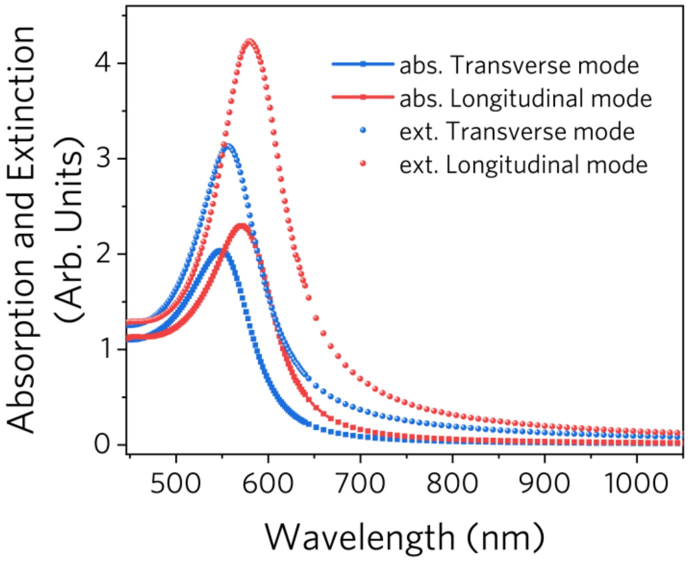

Figure S12. Absorption and extinction spectra of different plasmonic resonance modes.

600 nm.

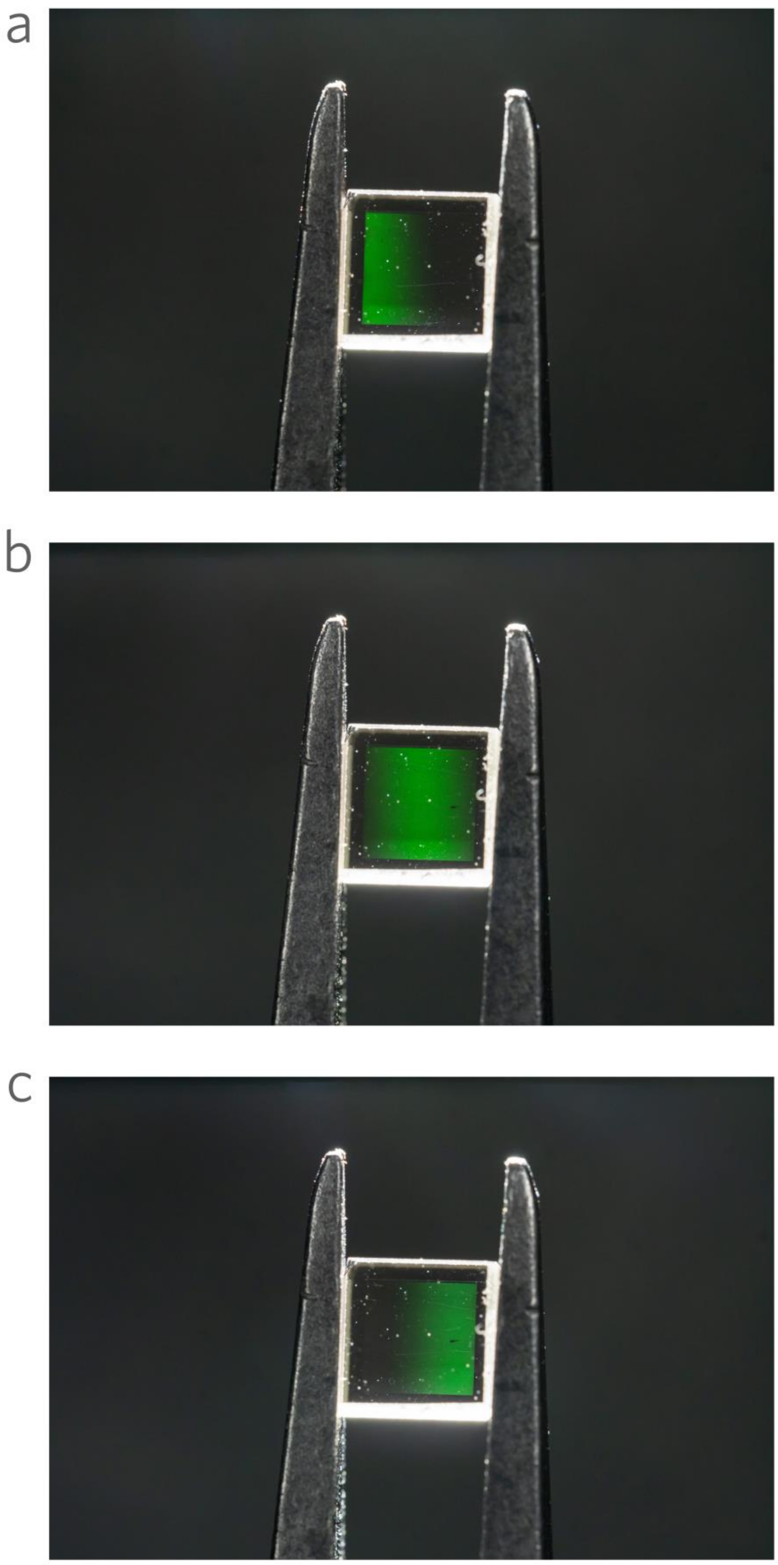

Figure S13. Camera-captured transmitted structural color of the nanograting with a period of 600 nm.

Supplementary References

1. http://www.srim.org/ (accessed: October 2021).
2. H. Zhu, M. Gao, C. Pang, R. Li, L. Chu, F. Ren, W. Qin, F. Chen, *Small Sci.* **2022**, *2*, 2100094.
3. G. Rizza, Y. Ramjauny, T. Gacoin, L. Vieille, S. Henry, *Phys. Rev. B* **2007**, *76*, 245414.
4. V. Ramaswamy, T. E. Haynes, C. W. White, W. J. MoberlyChan, S. Roorda, M. J. Aziz, *Nano Lett.* **2005** *5*, 373.
